# Genetic associations and potential mediators between psychiatric disorders and irritable bowel syndrome: a Mendelian randomization study with mediation analysis

**DOI:** 10.3389/fpsyt.2024.1279266

**Published:** 2024-01-30

**Authors:** Tao Zhang, Yuzhu Chen, Xiaoang Li, Jindong Zhang, Liping Duan

**Affiliations:** Department of Gastroenterology, Peking University Third Hospital, Beijing, China

**Keywords:** psychiatric disorders, irritable bowel syndrome, Mendelian randomization, mediation analysis, causal effect

## Abstract

**Objective:**

Potential causal associations between psychiatric disorders and irritable bowel syndrome have been demonstrated in observational studies; however, these studies are susceptible to underlying confounding and reverse causation biases. We aimed to assess the causal effects of psychiatric disorders on irritable bowel syndrome (IBS) and the potential mediators from a genetic perspective by conducting a Mendelian randomization (MR) study with mediation analysis.

**Method:**

Genetic instruments associated with psychiatric disorders, potential mediators, and IBS were obtained from large-scale genome-wide association studies (GWAS). Three MR methods - the inverse-variance weighted (IVW) method, MR-Egger method, and weighted median method, were used to investigate causal association estimates. Heterogeneity among different genetic instrumental variables (IVs) was assessed using Q tests. Additionally, the MR-PRESSO and MR-Pleiotropy methods were used to verify horizontal pleiotropy and detect outliers that might bias the results, which were removed from further analysis. Consequently, we used MR mediation analysis to investigate potential mediators in the causal associations between psychiatric disorders and IBS.

**Results:**

MR provided evidence of the causal effects of genetically predicted broad depression, major depressive disorder (MDD), anxiety disorder, post-traumatic stress disorder (PTSD), and schizophrenia on IBS. The results of MR mediation analysis demonstrated that the reduction in acetate levels mediated 12.6% of the effects of broad depression on IBS; insomnia mediated 16.00%, 16.20%, and 27.14% of the effects of broad depression, MDD, and PTSD on IBS, respectively; and the increase in blood β-hydroxybutyrate levels mediated 50.76% of the effects of schizophrenia on IBS.

**Conclusion:**

Our study confirmed the brain-gut axis involvement and potential modulators in the pathophysiology of psychiatric disorder-induced IBS from a genetic perspective, and suggests potential therapeutic targets for the disrupted brain-gut axis.

## 1 Introduction

Irritable bowel syndrome (IBS) is a common and chronic gut-brain interaction disorder, characterized by a combination of abdominal symptoms such as pain, bloating, and changes in bowel habits and stool form, with an abnormal mental state (1, 2). IBS affects people across the world, with a reported prevalence of 7.0% in Southeast Asian and Middle Eastern studies, 11.8–14.0% in North American, Northern European, and Australian studies, and 15.0–21.0% in Southern European, African, and South American studies (3). Although IBS is not a life-threatening condition, it reduces the quality of life and imposes a high socioeconomic burden on patients, especially women and young individuals (4, 5). The pathophysiology of IBS is complex and not completely understood; however, genetics, diet, infectious gastroenteritis, and the gut microbiome are known risk factors for IBS (6). Genetic variants in genes encoding serotonin transporter (SLC6A4), sucrase-isomaltase or SCN5A (a voltage-gated sodium channel), and corticotropin-releasing hormone (CRH) receptors 1 and 2 may be associated with IBS (6).

Numerous studies have shown that psychiatric disorders frequently accompany IBS, particularly depressive and anxiety disorders, which occur in up to 23% of IBS cases (6, 7). Comorbidities of post-traumatic stress disorder (PTSD) and schizophrenia have also been identified in IBS patients; for instance, 36% of IBS patients met the lifetime diagnostic criteria of PTSD (8, 9), and 13.6% of PTSD patients met the Rome III criteria for IBS (9, 10). The prevalence of IBS in schizophrenia and attention-deficit/hyperactivity disorder (ADHD) was significantly higher than that in the control group without schizophrenia and ADHD (8, 11, 12). Interestingly, a recent genome-wide pleiotropic association study revealed a shared genetic etiology between IBS and PTSD, ADHD, bipolar disorder, major depressive disorder (MDD), and anorexia nervosa (AN), indicating that IBS and psychiatric disorders may share a common or linked pathogenic pathway in the brain-gut axis (13). According to previous studies, psychological symptoms and disorders may have developed as a result of the effect of IBS on an individual or may have existed before the onset of digestive symptoms (6, 14). All evidence suggests that the brain–gut axis (the bidirectional interaction between the central and enteric nervous systems) plays a pivotal role in the pathophysiology of IBS (15). However, despite strong epidemiological evidence on the association between IBS and psychiatric disorders, most studies have been observational, with limitations of being susceptible to potential confounding and reverse causation biases, which can lead to inconsistent and biased results. Consequently, the current understanding of the association between IBS and psychiatric disorders remains tenuous.

In this study, we aimed to explore whether psychiatric disorders are risk factors for IBS by conducting a Mendelian randomization (MR) study. MR is a widely used genetic epidemiological method that utilizes single nucleotide polymorphisms (SNPs) as instrumental variables (IVs) to investigate the potential causal effects of risk factors on health outcomes. Accumulating evidence has proven the reliability of MR studies. Alleles are randomly separated during meiosis; therefore, MR is less likely to be influenced by confounders (e.g., environmental exposure, socioeconomic status, and behavior). MR can also prevent the reverse causation bias that exists in observational findings because genetic variation occurs before the disease, and the order of the two cannot be reversed (16–18).

In difficult cases, such as the above-mentioned causal associations between psychiatric disorders and IBS, when MR findings are similar with those of observational studies and consistently demonstrate the associations, it provides a higher level of confidence in establishing the causal relationships between these two pathologies, and the interplay in the brain-gut axis can be further confirmed from a genetic perspective. This will prompt physicians to pay more attention to the digestive symptoms of patients with psychological distress at an earlier stage than before. It will facilitate a more proactive approach to managing and treating these disorders adequately to prevent psychotic episodes, exacerbation of digestive symptoms, and subsequent development of IBS. Ultimately, this approach will help to improve patients’ quality of life. In this study, we performed an MR mediation analysis to investigate the potential mediators between psychiatric disorders and IBS to explore the underlying pathophysiological modulators of brain-gut interactions.

## 2 Materials and methods

### 2.1 Data sources

#### 2.1.1 Psychiatric disorders

Two sets of genetic instruments were used to determine the cause of depression in patients with IBS. Fourteen independent variants significantly associated with broad depression (defined as self-reported past help-seeking for mental health difficulties such as nerves, anxiety, tension, or depression) were identified through a genome-wide association study (GWAS) meta-analysis conducted in 2018 with a participant pool of 322,580 UK Biobank participants (19). Genetic instruments for MDD were also derived from a GWAS meta-analysis performed in 2018, which contained seven cohorts with 135,458 MDD cases and 344,901 controls (20), in which 44 SNPs were reported to be significantly related to MDD.

Only five genome-wide SNPs significantly associated with lifetime anxiety disorders were selected from the 2019 GWAS (21), including 25,453 cases and 58,113 controls (21). Genetic instruments for PTSD were taken from the most recent GWAS by Wendt et al. (22), who reported 32 independent SNPs that showed genome-wide significance and found to be associated with PTSD when meta-analyzed using the 6-item PTSD trait. Genetic instruments for schizophrenia were obtained from a GWAS meta-analysis conducted in 2022 by Trubetskoy et al. (23), who identified 343 independent SNPs located at 287 loci in an extended GWAS. Thirty SNPs associated with bipolar disorder were obtained from a GWAS by Stahl et al. (24), who used data from 198,882 individuals of European ancestry collected from 39 cohorts across Europe, North America, and Australia.

In addition to the above-mentioned psychiatric disorders that are usually associated with IBS, we further explored several other psychiatric disorders, including Autism Spectrum Disorder (ASD) (25), ADHD (26), and AN (27), that show microbiota-gut-brain axis dysfunction. All sources of the SNPs are listed in **Table 1**. Furthermore, the inclusion and exclusion criteria of patients with psychiatric disorders were important to identify genome-wide-significant loci and can be found in these GWAS study or GWAS meta-analysis, especially the prescribed medication was considered as one of the key indicators, for example, the patients who reported having a prescribed medications for any psychiatric disorders were excluded when selecting healthy/control individuals (19, 21, 23, 24).

**Table 1:** Overview of databases used for gene-exposure and gene-outcome data.

Phenotype	Sample size	Ethnicity	PMID
**Broad depression**	322,580 UK Biobank participants	European	29662059
**MDD**	135,458 cases and 344,901 controls	European and American	29700475
**Anxiety disorder**	25,453 cases and 58,113 controls	European	31748690
**PTSD**	497,803 cases	European	35181757
**Schizophrenia**	76,755 cases and 243,649 controls	European, East Asian, African-American, Latino	35396580
**Bipolar disorder**	29,764 cases and 169,118 controls	European	31043756
**ASD**	18,381 cases and 27,969 controls	European	30804558
**ADHD**	20,183 cases and 35,191 controls	European, American, Asian	30478444
**AN**	16,992 cases and 55,525 controls	European	31308545
**Gut microbiota**	18,340 individuals	European, Middle-Eastern, East Asian, American Hispanic/Latin, African American, etc.	33462485
**LPS**	11,296 individuals	European	34668383
**Tryptophan**	86,507 individuals	European	33414548
**Histamine, Acetate, Lactate, Pyruvate, and β-Hydroxybutyrate**	115,082 individuals	European	35213538
**CRP**	418,642 participants	European	31900758
**IL-6**	67,428 individuals	European	33517400
**BDNF**	11,785 individuals	European	33345186
**Catalase**	1,000 individuals	European	28240269
**Sleep duration**	446118 individuals	European	30846698
**Insomnia**	129,270 cases and 108,357 controls	European	30804566
**BMI**	700,000 participants	European	30124842
**IBS**	53,400 cases and 433,201 controls	European	34741163

LPS, lipopolysaccharide; CRP, C-reactive protein; BDNF, brain-derived neurotrophic factor.

#### 2.1.2 Potential mediators

The term “brain–gut–microbiome axis” has long been proposed, whereby the composition and function of gut microbiota are affected by the abnormal central nervous system, resulting in gastrointestinal symptoms due to the related dysbiosis intestinal homeostasis, such as inflammation, oxidative stress, as well as changes in microbiome-derived neurotransmitters, metabolites, neuroendocrine factors, and enzymes (28). However, details of the microbiome-related pathophysiology of IBS remain elusive. Additionally, sleep disorders and an increase in body mass index (BMI) are triggered by psychiatric stressors (29, 30) and are also risk factors for IBS (31, 32); however, whether BMI and sleep disorders mediate the causal effects between psychiatric disorders and IBS remains unclear. In this study, we suspected that these factors are potential mediators of the causal effects of psychiatric disorders on IBS. Genetic instruments of the gut microbiota were acquired from a large-scale association study (33) containing 24 cohorts of 18,340 participants from different ancestries, most of whom were European. The sources of the IVs for the other potential mediators are shown in **Table 1** (34–43).

#### 2.1.3 IBS

Genetic instruments associated with IBS were derived from the latest GWAS conducted by Eijsbouts et al. (44) encompassing 53,400 European cases and 433,201 controls. These IBS cases should meet at least one of the following four conditions: 1) meet the digestive health questionnaire (DHQ) Rome III symptom criteria without other diagnostic explanations for these symptoms; 2) have a DHQ self-report of previous medical IBS diagnosis or electronic medical records; 3) provide an unprompted ‘self-report’; and 4) a self-reported IBS diagnosis in response to the question ‘Has a doctor ever told you that you have any … serious medical conditions?’ Linked hospital episode statistics indicated that hospital admission due to IBS was the primary or secondary ICD-10 diagnosis. The DHQ also asked about previous IBS diagnosis, environmental exposures and associated conditions (including anxiety or depression, based on treatment sought or offered). Full GWAS summary statistics are available at https://www.ebi.ac.uk/gwas/publications/34741163.

### 2.2 Mendelian randomization design and SNP selection

**Figure 1:**
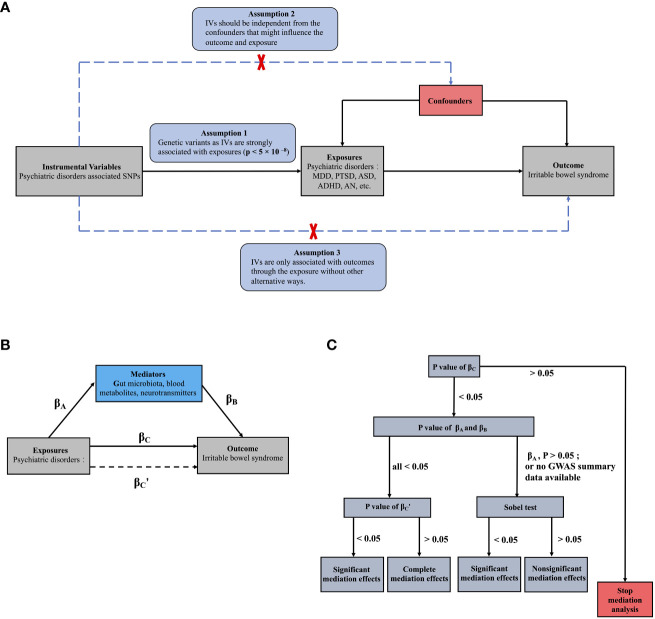
The main design of this MR study: **(A)** Three assumptions of the MR study; **(B)** Basic schematic of mediation analysis; **(C)** The process of MR mediation analysis in the present study. **(B)** β_A_ represents the regression coefficients for the association between psychiatric disorders and potential mediators; β_B_ represents the regression coefficients for the association between potential mediators and IBS; β_C_ represents the total effect between psychiatric disorders and IBS, without the adjustment for mediator; and β_C_’ represents the direct effect between psychiatric disorders and IBS, taking into account adjustment for potential mediators.

The MR is established based on three core assumptions, as shown in **Figure 1A**: (i) relevance: IVs such as SNPs are strongly associated with exposures (p < 5 × 10^–8^); (ii) independence: instrumental SNPs should be independent from the potential confounders that might influence the outcome and exposures; and (iii) exclusion hypothesis: SNPs are only associated with outcome through the exposures without other alternative ways, that is, if SNPs can directly affect the outcomes without through the exposure, the results of MR have horizontal pleiotropic effects and violated the third assumption (18). The most important step in MR analysis is the selection of the appropriate SNPs to be used as instruments (**Supplementary Figure 1**), which must be strongly linked to exposures with low linkage disequilibrium (LD) (r^2^ < 0.001, window size = 10000 kb). To meet the second assumption and reduce interference from confounders in *Phenoscanner* (http://www.phenoscanner.medschl.cam.ac.uk/), each instrumental SNP was estimated for possible connections with confounders, including BMI, white matter microstructure, sleep duration, insomnia, household income, and smoking. SNPs associated with these confounders were subsequently excluded from MR analysis. For harmonization, palindromic SNPs were excluded because the directions of the positive and negative chains could not be determined for the same alleles on both strands.

We used univariable two-sample MR study to investigate the causal associations between psychiatric disorders and IBS. Then we further investigated potential pathways from psychiatric disorders to IBS by using univariable and multivariable MR studies: First, the causal effects of the potential mediators proposed in *Part 2.1.2* on IBS were investigated through a univariable two-sample MR study (β_B_). Second, the causal associations between psychiatric disorders and these mediators that showed significant causal effects on IBS were also acquired (β_A_). Lastly, MR mediation analysis was utilized to investigate whether the mediators associated with both psychiatric disorders (β_A_; P < 0.05) and IBS (β_B_; P < 0.05) mediated any causal effects of psychiatric disorders on IBS (45). For the mediation analysis, multivariable MR was performed as previously described (46). Briefly, IVs for both psychiatric disorders and mediators were included in the same MR model to investigate their direct effects on IBS after mutual adjustment. Any attenuation of the direct MR effects (β_C_’; P<0.05) of psychiatric disorders, after adjusting for mediators, would support mediation through mediators (**Figures 1B, C**) compared with those observed in the two-sample MR measuring the total effects (β_C_) of psychiatric disorders on IBS (47, 48). Additionally, if the β_A_ showed nonsignificant P values or the GWAS summary data for psychiatric disorders or mediators were not fully available, limiting multivariable MR analysis, the Sobel test was used to infer mediation effects (49).

### 2.3 Statistical methods

All statistical analyses were performed using R 4.1.0 with the R packages “TwoSampleMR”, “MR-PRESSO”, and “MRlap”. In the MR estimation, we chose the inverse-variance weighted (IVW) method as the primary analysis, which can obtain an unbiased result only when all IVs are valid with no directional pleiotropy effects. MR analysis was also conducted using other methods to check the robustness of the results, such as the weighted median method and MR-Egger method, which can estimate the causal effect in cases where some IVs are invalid (31). The weighted median method estimates allow up to 50% of the information in the analysis to come from invalid IVs; thus, it provides consistent effect estimates even in the presence of IVs with heterogeneous effects (50). Considering pleiotropic effects, the MR-Egger method provides a valid causal estimate if the SNP-exposure associations are not correlated with the direct effects of IVs on the outcome (51). If the P values of MR estimation were less than 0.05, it indicates significant causal associations. Additionally, the MR-pleiotropy method was used to verify the horizontal pleiotropy, and the MR-Pleiotropy Residual Sum and Outlier method (MR-PRESSO) was used to detect outliers that might introduce bias into the results. Subsequently, the outliers were removed from further analyses. In addition, the presence of heterogeneity among different IVs was assessed using Cochran’s (IVW) and Rücker’s (MR-Egger) Q tests.

Given that our GWAS summary statistics are mostly drawn from European populations; the possibility of sample overlap cannot be ignored. To mitigate potential bias arising from sample overlap, we applied the MRlap function to correct the IVW results (52). If the corrected effects are closely consistent with the observed effects and do not exhibit significant differences, then we can be reasonably confident in the IVW-MR estimates. Conversely, if substantial differences occur, the corrected effect should be given priority because it is independent of sample overlap. GWAS summary data on exposures and outcomes were needed in MRlap analysis, and if it was not available, we assessed the bias and Type 1 error rates caused by sample overlap in MR (53).

## 3 Results

### 3.1 Step 1: Associations between genetically predicted psychiatric disorders and IBS

**Figure 2:**
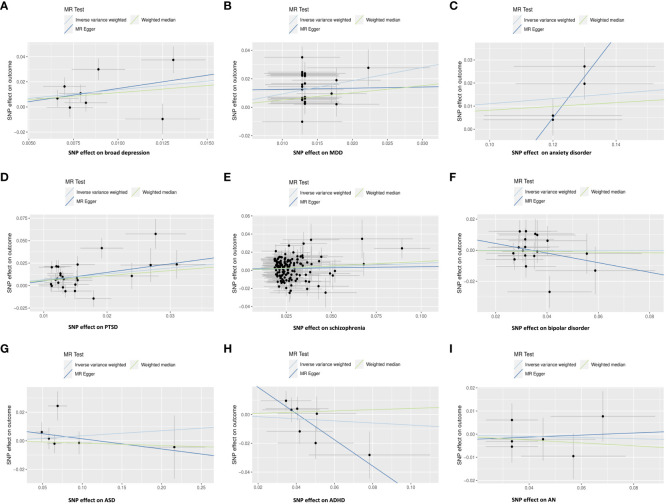
Scatter plot: Relationships between genetically predicted psychiatric disorders and IBS using the IVW method, weighted median method, and MR-Egger method. **(A)** broad depression; **(B)** MDD; **(C)** anxiety disorder; **(D)** PTSD; **(E)** schizophrenia; **(F)** bipolar disorder; **(G)** ASD; **(H)** ADHD; **(I)** AN.

**Figure 3:**
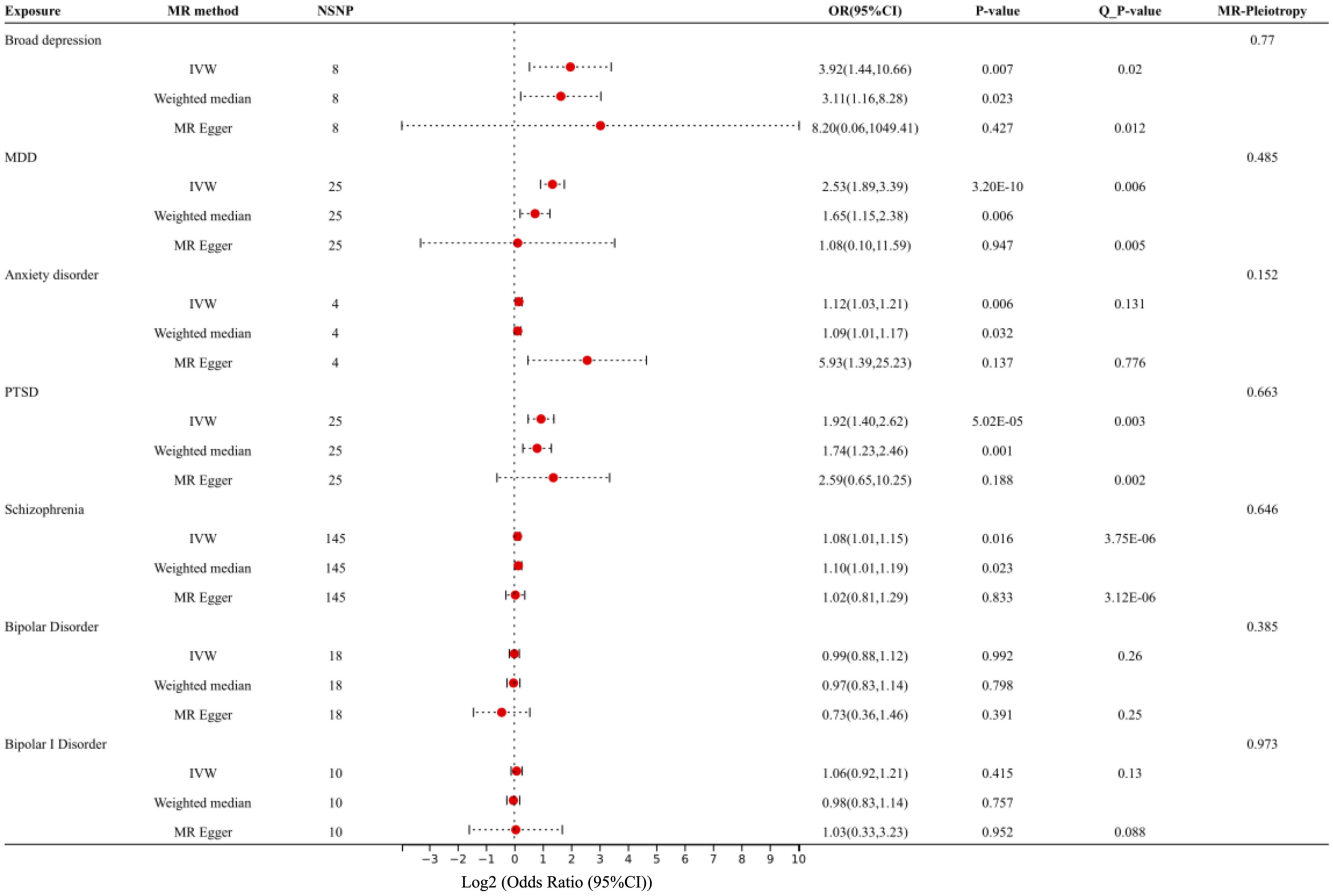
Forest plot: Association of genetically predicted broad depression, MDD, anxiety, PTSD, schizophrenia, bipolar disorder, and IBS, using the IVW method, weighted median method, and MR-Egger method. The effect estimates are presented as odds ratios (OR, odds ratios; NSNP, the number of single nucleotide polymorphisms; 95% CI, 95% confidence interval; Q_P value, the results of the Q test).

**Figure 4:**
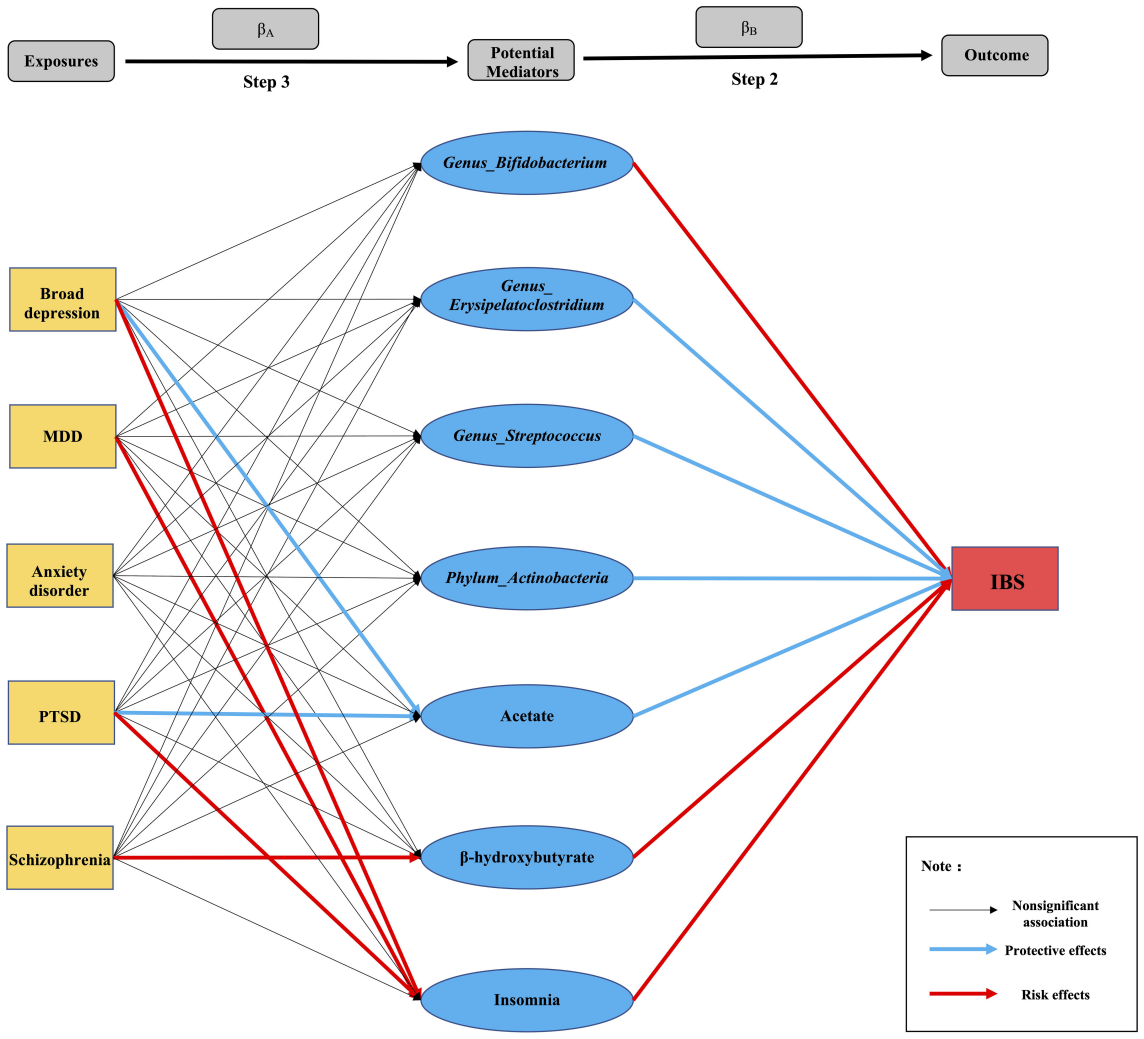
Summary diagram of Step 2 and Step 3: Each line segment between exposures and outcomes represents a connection, and the arrow represents the direction of the association.

After removing the SNPs in LD or palindromes, the outliers identified in the MR-PRESSO method, as well as the SNPs associated with confounders between psychiatric disorders and IBS (i.e., broad depression: white matter abnormalities (54, 55); MDD: sleep disorders (56–58); PTSD: household income (59, 60); schizophrenia: insomnia, smoking, and white matter abnormalities (61–64); bipolar disorder: BMI (65, 66); ADHD: insomnia (67)), the remaining SNPs utilized as IVs are shown in **Supplementary Tables S1-S9**. Both the IVW MR method and weighted median method showed statistically significant associations between genetically predicted broad depression (IVW OR: 3.92, P: 0.007; weighted median OR: 3.11, P:0.023), MDD (IVW OR: 2.53, P: 3.20×10^-10^; weighted median OR: 1.65, P: 0.006), anxiety disorder (IVW OR: 1.12, P: 0.006; weighted median OR: 1.09, P: 0.032), PTSD (IVW OR: 1.92, P: 5.02×10^-5^; weighted median OR: 1.74, P: 0.001), schizophrenia (IVW OR: 1.08, P: 0.016; weighted median OR: 1.10, P: 0.023) and risk of IBS, although the MR-Egger method showed no statistical significance. The MR pleiotropy method identified non-significant horizontal pleiotropy in these associations, while the Q tests showed significant heterogeneity except for anxiety disorders (**Figures 2A–E**, **3**). Moreover, non-significant associations were found between genetically predicted bipolar disorder, bipolar I disorder, ASD, ADHD, AN, and the risk of IBS using all three methods (P > 0.05), and neither heterogeneity nor horizontal pleiotropy was identified in these associations, suggesting that these results were consistent (**Figures 2F–I**, **3**, **4**).

### 3.2 Step 2: Associations between genetically predicted potential mediators and IBS

We investigated 24 potential mediators of the causal effects of psychiatric disorders on IBS after a literature review. As shown in **Supplementary Table S10**, the gut microbiota genera, *Erysipelatoclostridium* (IVW OR: 0.895, P =0.028) and *Streptococcus* (IVW OR: 0.958, P: 0.002) had protective effects on IBS. However, the causal effects of the *Actinobacteria* phylum and its genus *Bifidobacterium* on IBS showed inconsistent results, i.e., the estimates of the IVW method and weighted median method showed the protective effects of the *Actinobacteria* phylum (IVW OR:0.944, P: 1.826E-5) and *Bifidobacterium* genus (IVW OR: 0.982, 0.045) on IBS, while the MR-Egger method showed anti-protective effects (*Actinobacteria* phylum, MR-Egger OR: 1.348, P: 0.046; *Bifidobacterium* genus, MR-Egger OR: 1.179, P: 0.002). No causal associations were found between IBS and the genera *Allisonella*, *Enterorhabdus*, *Ruminococcus1*, *Ruminococcustorques*, and *Tyzzerella3*.

Regarding blood metabolites, inflammatory biomarkers, neurotransmitters, and acetate showed protective effects on IBS (IVW OR: 0.719, P: 0.048); however, β-hydroxybutyrate showed anti-protective effects (weighted median, OR: 1.294, P: 0.004). Simultaneously, inconsistent results were also observed in the association between IL-6 and IBS, in which the IVW (OR: 1.137, P: 6.923E-16) and weighted median method (OR: 1.069, P: 0.009) showed that increasing levels of IL-6 had a causal effect on IBS, whereas, the MR-Egger method showed that IL-6 had a protective effect on IBS (OR:0.883, P =0.023). Additionally, there was no evidence supporting the causal associations among genetically predicted lipopolysaccharide (LPS), C-reactive protein (CRP), tryptophan, histamine, lactate, pyruvate, catalase, or brain-derived neurotrophic factor (BDNF) and IBS in any of the three methods of our MR study.

Insomnia was a significant risk factor for IBS (IVW OR: 2.051; P: 1.548E-5), as shown by all three MR methods. However, there was no significant causal relationship between sleep duration and IBS (IVW OR: 0.916; P: 0.740). Furthermore, we found no significant association between genetically predicted BMI and IBS using the three MR methods (IVW OR: 1.056, P:0.994) (**Supplementary Tables S11-S34**).

### 3.3 Step 3: Associations between genetically predicted psychiatric disorders and potential mediators significantly associated with IBS

Here, we assumed that psychiatric disorders with significant causal associations with IBS were exposures (broad depression, MDD, anxiety disorder, PTSD, and schizophrenia), and potential mediators with significant causal associations with IBS were outcomes to explore their causal associations (β_A_). We found that broad depression (IVW OR: 0.598, P: 0.031) and PTSD (IVW OR: 0.867, P: 0.021) causally led to lower levels of acetate and an increased risk of insomnia (broad depression, IVW OR: 1.356, P: 0.001; PTSD, IVW OR: 1.279, P: 4.085E-10). In addition to broad depression and PTSD, an increased risk of insomnia was found in MDD (IVW OR: 1.234, P: 1.397E-07); however, all showed significant heterogeneity. Furthermore, the MR-Egger method showed a significant causal effect of genetically predicted schizophrenia on increased blood β-hydroxybutyrate levels (IVW OR: 1.164, P:0.007), with significant horizontal pleiotropy (P = 0.015). Unfortunately, since the GWAS summary data for IL-6 were not available, we were unable to study the causal effects of psychiatric disorders on IL-6 levels, and we did not find any potential mediators affected by anxiety disorders, which requires further exploration (**Supplementary Table S35**).

### 3.4 Step 4: MR mediation analysis

Based on the results of step 2 and step 3 (**Figure 4**), we conjectured that 1) the reduction in acetate might mediate the effects of broad depression and PTSD on IBS; 2) insomnia might mediate broad depression, MDD, and PTSD-induced IBS; and 3) the increase in β-hydroxybutyrate levels in the blood might mediate schizophrenia-induced IBS (P values of β_A_, β_B_, and β_C_ were all lower than 0.05). Thus, we performed a multivariable MR to investigate β_C_’. We found a significant attenuation in the effect of genetically predicted broad depression on IBS after adjusting for acetate or insomnia (β_C_’, P < 0.05), which mediated 12.60% and 16.00% causal effects of broad depression on IBS, respectively. Unfortunately, since the GWAS summary data for MDD, PTSD, and schizophrenia were not fully available, we were unable to perform multivariate MR analysis to identify their mediators. Therefore, Sobel tests were performed instead, in which we verified that insomnia mediated 16.20% and 27.14% of the causal effect of genetically predicted MDD and PTSD on IBS, respectively, and the increase in β-hydroxybutyrate levels in the blood mediated 50.76% of the causal effect of genetically predicted schizophrenia on IBS (**Table 2**).

**Table 2:** The results of MR mediation analysis.

Exposure	mediator	β_A_	S_A_	β_B_	S_B_	β_C_	β_C_’	Sobel test (P-value)	Mediation effects
Broad depression									
	***Genus_Bifidobacterium***	0.287	0.544	**0.164^#^**	0.052	**1.368**	NA	0.603	NO
	***Genus_Erysipelatoclostridium***	-0.285	0.664	**-0.111**	0.051		NA	0.674	NO
	***Genus_Streptococcus***	-0.313	0.546	**-0.043**	0.014		NA	0.573	NO
	***Phylum_Actinobacteria***	0.927	0.513	**-0.057**	0.013		NA	0.096	NO
	**Acetate**	**-0.514**	0.239	**-0.330**	0.167		**1.162**	NA	**12.60%**
	**β-Hydroxybutyrate**	-0.051	0.186	**0.257^#^**	0.090		NA	0.785	NO
	**Insomnia**	**0.304**	0.095	**0.719**	0.190		**0.787**	N/A	**16.00%**
MDD									
	***Genus_Bifidobacterium***	0.053	0.177	**0.164^#^**	0.052	**0.932**	NA	0.767	NO
	***Genus_Erysipelatoclostridium***	-0.069	0.224	**-0.111**	0.051		NA	0.761	NO
	***Genus_Streptococcus***	0.010	0.168	**-0.043**	0.014		NA	0.955	NO
	***Phylum_Actinobacteria***	-0.077	0.161	**-0.057**	0.013		NA	0.180	NO
	**Acetate**	0.069	0.062	**-0.330**	0.167		NA	0.336	NO
	**β-Hydroxybutyrate**	-0.032	0.089	**0.257^#^**	0.090		NA	0.723	NO
	**Insomnia**	**0.210**	0.040	**0.719**	0.190		NA	**0.002**	**16.20%**
Anxiety disorder									
	***Genus_Bifidobacterium***	0.006	0.045	**0.164^#^**	0.052	**0.040**	NA	0.900	NO
	***Genus_Erysipelatoclostridium***	-0.039	0.055	**-0.111**	0.051		NA	0.501	NO
	***Genus_Streptococcus***	-0.037	0.043	**-0.043**	0.014		NA	0.404	NO
	***Phylum_Actinobacteria***	0.073	0.056	**-0.057**	0.013		NA	0.210	NO
	**Acetate**	0.000	0.020	**-0.330**	0.167		NA	0.997	NO
	**β-Hydroxybutyrate**	-0.005	0.028	**0.257^#^**	0.090		NA	0.854	NO
	**Insomnia**	0.007	0.008	**0.719**	0.190		NA	0.365	NO
PTSD									
	***Genus_Bifidobacterium***	-0.107	0.190	**0.164^#^**	0.052	**0.651**	NA	0.580	NO
	***Genus_Erysipelatoclostridium***	0.143	0.242	**-0.111**	0.051		NA	0.567	NO
	***Genus_Streptococcus***	0.054	0.197	**-0.043**	0.014		NA	0.784	NO
	***Phylum_Actinobacteria***	-0.247	0.211	**-0.057**	0.013		NA	0.259	NO
	**Acetate**	**-0.143**	0.062	**-0.330**	0.167		NA	0.134	NO
	**β-Hydroxybutyrate**	0.094	0.064	**0.257^#^**	0.090		NA	0.191	NO
	**Insomnia**	**0.246**	0.039	**0.719**	0.190		NA	**0.001**	**27.14%**
Schizophrenia									
	***Genus_Bifidobacterium***	-0.042	0.047	**0.164^#^**	0.052	**0.077**	NA	0.390	NO
	***Genus_Erysipelatoclostridium***	0.035	0.055	**-0.111**	0.051		NA	0.542	NO
	***Genus_Streptococcus***	0.043	0.040	**-0.043**	0.014		NA	0.310	NO
	***Phylum_Actinobacteria***	-0.025	0.044	**-0.057**	0.013		NA	0.573	NO
	**Acetate**	0.030	0.016	**-0.330**	0.167		NA	0.177	NO
	**β-Hydroxybutyrate**	**0.152^#^**	0.055	**0.257^#^**	0.090		NA	**0.047**	**50.76%**
	**Insomnia**	-0.003	0.008	**0.719**	0.190		NA	0.684	NO

S_A_, standard error of β_A_; S_B_, standard error of β_B_;

The bold β values in this table indicate that their P values are less than 0.05;

^#^: β values from MR-egger method or Weighted median method; while in this table, the other values were from IVW method.

NA, not available.

NO, no mediation effects.

### 3.5 MRlap analysis

We found that the samples in GWAS on broad depression, MDD, anxiety disorder, PTSD, AN, blood metabolites, CRP, sleep duration, insomnia, and BMI might be overlapped with the samples from GWAS on IBS, because they all contained the participants from UK Biobank. The GWAS summary data on broad depression, blood metabolites (acetate, lactate, pyruvate, and β-hydroxybutyrate), sleep durations, insomnia, and BMI were available, thus the MRlap analysis was performed, which showed that the causal effects of these indicators on IBS, as determined by the MRlap correction, align with those obtained through the primary MR analyses (**Supplementary Table S36**). The GWAS summary data on MDD, anxiety disorder, PTSD, AN, histamine, and CRP were not available in our included studies, however, low bias and Type 1 error rates were found (**Supplementary Table S37**). These outcomes reaffirmed the robustness of the IVW method.

## 4 Discussion

### 4.1 MR study confirmed the causal associations between psychiatric disorders and IBS

To the best of our knowledge, this is the first study to illustrate the causal associations and potential mediators between psychiatric disorders and IBS from a genetic perspective. In this two-sample MR study, we found that broad depression, MDD, lifetime anxiety disorder, schizophrenia, and PTSD were risk factors for IBS.

Depression and anxiety are common psychiatric symptoms of IBS. In a Chinese population, diarrhea-predominant IBS (IBS-D) and depressive disorder showed two shared genetic variants (*SYT8* rs3741231 G allele and *SSPO* rs12536873 TT genotype) associated with neurogenesis and neurotransmission, providing a genetic basis for the high comorbidity of IBS-D and depressive disorder (68). A recent meta-analysis showed that patients with IBS have an eight-fold greater risk of anxiety and a seven-fold greater risk of depression than controls in the Indian population (69). Two prospective studies have found that higher levels of anxiety and depression at baseline were significant predictors of IBS at the 1-year and 12-year follow-ups; conversely, IBS patients without anxiety and depression at baseline reported significantly higher levels of anxiety and depression at the 1-year follow-up, suggesting that independent gut-brain and brain-gut pathways operate in the pathophysiology of IBS (15, 70). However, it is likely that underlying confounders, such as changes in medication, life stressors, environmental exposure, socioeconomic burden, lifestyle, or other conditions unrelated to gastroenterology, will affect these data over a long or even a short period of time, leading to biased results (70). MR studies can methodologically exclude interference from these confounders. The results of our study agree with those of these longitudinal studies, further confirming the above evidence.

The association between PTSD and IBS remains controversial. A meta-analysis (10) comprising eight studies showed that PTSD was a significant risk factor for IBS. This finding is consistent with another study, which revealed a correlation between early-life abuse and trauma and a higher risk for the development of IBS in adulthood (8). However, some studies have disputed this association, as they found that a history of severe sexual/physical abuse was associated with higher pain thresholds for rectal distension in women with IBS (71). Additionally, a single-arm study showed that the prevalence of PTSD was not higher among patients with IBS than in the general population (72). Interestingly, our study confirmed that genetically predicted PTSD is a risk factor for IBS, opening new directions for future mechanistic studies.

Schizophrenia is a complex and debilitating brain disorder characterized by behavioral abnormalities, including cognitive dysfunction, psychosis, delusions, apathy, and withdrawal (73). Some risk factors for schizophrenia, such as inflammation, food intolerance, and *Toxoplasma gondii* exposure, partly involve the biological pathways of the gut, indicating that the gut-brain interaction may play a pivotal role in the pathophysiology of schizophrenia (73). To date, only a few studies have explored the association between schizophrenia and IBS, and meta-analyses have been conducted to verify this relationship. A cohort study by Lee et al. (74) showed no statistically significant difference in the incidence rate of schizophrenia between the IBS cohort and the cohort without IBS, even after 5 years of follow-up. Another small-scale retrospective study in 1997 suggested that the schizophrenia group exhibited a higher risk of IBS than the control group (11). After over 25 years, this result was reconfirmed by our MR study.

### 4.2 MR study suggests no causal link between bipolar disorder and IBS.

Abnormal levels of cytokines, including TNF-α, IL-8, and IL-10, are significantly associated with IBS and bipolar disorder symptoms, indicating that IBS and bipolar disorder share similar pathophysiological mechanisms (75–79). Thus, the comorbidity of both disorders was hypothesized. However, our MR study suggests no causality between bipolar disorder (including bipolar I disorder) and IBS incidence, which is consistent with the results of a meta-analysis of 11 studies that provided data on IBS (80). Conversely, IBS was found to increase the incidence of subsequent bipolar disorder in a nationwide cohort study; however, this finding needs to be further confirmed in future studies (74).

### 4.3 Possible factors mediating psychiatric disorder-induced IBS

Prior to the mediation analysis, we explored the causal associations between genetically predicted psychiatric disorders and potential mediators that are significantly associated with IBS. The findings indicated that broad depression and PTSD causally led to lower levels of acetate, as well as an increased risk of insomnia. Additionally, MDD was found to causally increase the risk of insomnia, while schizophrenia causally increased blood β-hydroxybutyrate levels. Therefore, acetate, insomnia, and β-hydroxybutyrate might play crucial roles in psychiatric disorders-induced IBS.

Acetate, the most abundant short chain fatty acid (SCFA) produced by the gut microbiota and a precursor used by many gut commensals to produce propionate and butyrate, affects the metabolic pathway through the G protein-coupled receptor and free fatty acid receptor 2 in colonic cells to improve bowel function (81, 82). A reduction in acetate, propionate, and butyrate levels was found in patients with IBS and linked to specific IBS symptoms such as colonic hyperalgesia and hypersensitivity (83). Additionally, a study showed that acetate supplementation produces antidepressant-like effects by increasing histone acetylation and improving synaptic plasticity in the hippocampus (84). Interestingly, in our MR mediation analysis, we found that the reduction in blood acetate mediated 12.6% of the causal effects of broad depression on IBS, which further supports the key beneficial role of acetate in the brain-gut axis. Unfortunately, in our MR study, although PTSD was causally associated with lower levels of acetate, which was a risk factor for IBS, we found no mediating effects of acetate on PTSD-induced IBS in the mediation analysis.

Another blood metabolite, β-hydroxybutyrate, a ketone body that serves as an energy source during starvation or exercise, shows several beneficial effects in the treatment of seizures, hypertension, NLRP3-mediated inflammation, and neurodegenerative diseases (85). The correlation between β-hydroxybutyrate and IBS has not been documented; however, in the present MR study, β-hydroxybutyrate showed anti-protective effects on IBS. Huang et al. (86) found that patients with schizophrenia had significantly higher serum levels of β-hydroxybutyrate than healthy controls, which was significantly correlated with fasting glucose and triglycerides, thus speculating that serum levels of β-hydroxybutyrate may act as a potential indicator of energy utilization impairment in schizophrenia. Consistently, our study showed that genetically predicted schizophrenia was a risk factor for increasing the β-hydroxybutyrate level in the blood, which further mediated 50.76% of total effects of schizophrenia on IBS.

Emerging evidence suggests that insomnia is positively correlated with many psychiatric disorders, such as ADHD, bipolar disorder, PTSD, MDD, OCD, and schizophrenia; bidirectional causal associations between insomnia and psychiatric disorders have also been observed (87), which might be explained by shared common abnormalities in hypothalamic–pituitary–adrenal (HPA) axis activation, serotonin system dysfunction, and overexpression of immune system peptides (88). A Korean population-based cohort study (56) found that subjects with insomnia also showed a higher prevalence of IBS than those without insomnia, which further highlights the pivotal role of insomnia in brain-gut axis disturbances. In the present study, we found significant causal effects of genetically predicted broad depression, MDD, and PTSD on insomnia, which is consistent with the results of a previous study (87). Insomnia was also a risk factor for IBS, mediating proportion of 16.00%, 16.20%, and 27.4% of the total effects of broad depression, MDD, and PTSD on IBS, respectively.

Overall, our study revealed that acetate, β-hydroxybutyrate, and insomnia were important mediators in psychiatric disorders-induced IBS, supporting their key roles in brain-gut axis. Discovering the key mediators in the causal association between two diseases can not only help to reveal the mechanisms, but also identify markers of disease evolution or exacerbation, so as to facilitate early intervention. For instance, our study found blood β-hydroxybutyrate level mediated schizophrenia-induced IBS, suggesting β-hydroxybutyrate as a potential biomarker. If the blood β-hydroxybutyrate level is elevated in patients with schizophrenia, IBS is likely to be induced, which will worsen abdominal symptoms and further reduce the patients’ quality of life. Therefore, early intervention to improve blood β-hydroxybutyrate level and mental health is important. Of course, these outcomes need further validations by more studies.

### 4.4 Strengths of this study

Overall, our study has several strengths. First, in observational settings, MR studies can simulate randomized controlled trials, which are costly, laborious, and time-consuming. In contrast, MR studies can technically reduce cost and effort and efficiently avoid confounding bias for SNPs that are randomly assigned at conception (89). Second, compared to other observational studies, MR studies can avoid the reverse causal effect and are less likely to be affected by confounders between exposure and outcome. Third, we selected significant genome-wide SNPs for diseases in the GWAS, which provided large and repeatedly checked samples. After a rigorous process of removing outliers, SNPs in LP or palindromes, and SNPs associated with underlying confounders, our study was more reliable. Fourth, we focused on nine psychiatric disorders with a high or low probability of comorbidity with IBS and further verified the causal association between them at the genetic level, filling the gap of insufficient research on the relationship between IBS and certain psychiatric disorders, such as bipolar disorder, schizophrenia, ASD, ADHD, and AN. Fifth, the utilization of mediation analysis and the multivariable MR method to identify potential mediators between psychiatric disorders and IBS is conducive to identifying the mechanisms of gut-brain axis abnormalities, which may improve health policies for managing these patients. Lastly, although sample overlap between GWAS summary data on exposures and outcomes in the analysis was inevitable, the results of MRlap analysis as well as low bias and Type 1 error rates in MR indicated the bias induced by sample overlap should be minimal in these causal estimates.

### 4.5 Limitations of this study

Although the present MR study was rigorously performed and had several strengths, some limitations remain. First, to minimize spurious causal relationships induced by individuals with diverse genetic backgrounds, the IVs in our study were selected from sources of European ancestry and genomic control research; thus, our results may not be generalizable to other races. Second, the heterogeneity in some results was challenging to eliminate; thus, we used three methods in addition to the IVW analysis to test the robustness of the results. Third, owing to the lack of a GWAS exploring the subtypes of IBS, we were unable to study the causal associations of these mental disorders with different types of IBS. Forth, the prevalence rate of IBS was higher in women than in men (5.2% vs. 2.9% in Rome-IV) and increasing evidence suggested the pathogenesis of IBS might differed by genders (90). However, since there were no GWAS summary data on IBS by genders, exploring the causal association of psychiatric disorders with IBS in different genders cannot be achieved through MR study. Furthermore, many gut microbiota, blood metabolites, and neurotransmitters are essential in mediating brain-gut axis communication. However, due to limited GWAS summary data, we could only explore the mediation role of some of them in psychiatric disorder-induced IBS; thus, larger, higher-quality, and more detailed GWASs are needed.

## 5 Conclusions

In the present MR study, we further verified that psychiatric disorders such as broad depression, MDD, lifetime anxiety disorder, schizophrenia, and PTSD could increase the risk of IBS. Furthermore, the MR mediation analysis demonstrated that the reduction in acetate levels mediated the effects of broad depression on IBS; insomnia mediated broad depression, MDD, and PTSD-induced IBS; and the increase in blood β-hydroxybutyrate levels mediated schizophrenia-induced IBS.

## Data availability statement

The original contributions presented in the study are included in the article/**Supplementary Material**. Further inquiries can be directed to the corresponding author.

## Author contributions

TZ: Conceptualization, Data curation, Formal analysis, Investigation, Methodology, Software, Writing – original draft. YC: Conceptualization, Formal analysis, Investigation, Methodology, Software, Writing – review & editing. XL: Conceptualization, Methodology, Software, Validation, Writing – review & editing. JZ: Conceptualization, Methodology, Validation, Visualization, Writing – review & editing. LD: Conceptualization, Funding acquisition, Supervision, Writing – review & editing.

## Supplementary material

Supplementary Figure 1 The screening and validation process of SNPs: Before initial MR analysis, the candidate SNPs that strongly associated with exposures, not associated with confounders, and with low linkage disequilibrium, were included. In the process of initial MR analysis, for harmonization, the palindromic SNPs were excluded. Subsequently, the horizontal pleiotropic effects were examined to validate whether SNPs can directly affect the outcomes without through the exposure so as to violate the third assumption. Lastly, the outliers that might introduce bias into the results were detected and excluded. After first round of exclusion of ineligible SNPs, the remained SNPs were included for a new round of MR analysis until all ineligible SNPs were excluded, and ultimately the final MR study were performed.
