# Supplementary figures and images for: Genetic associations and potential mediators between psychiatric disorders and irritable bowel syndrome: a Mendelian randomization study with mediation analysis
Source: Front Psychiatry. 2024 Jan 30;15:1279266. doi: 10.3389/fpsyt.2024.1279266 (PMC10861787; doi:10.3389/fpsyt.2024.1279266)

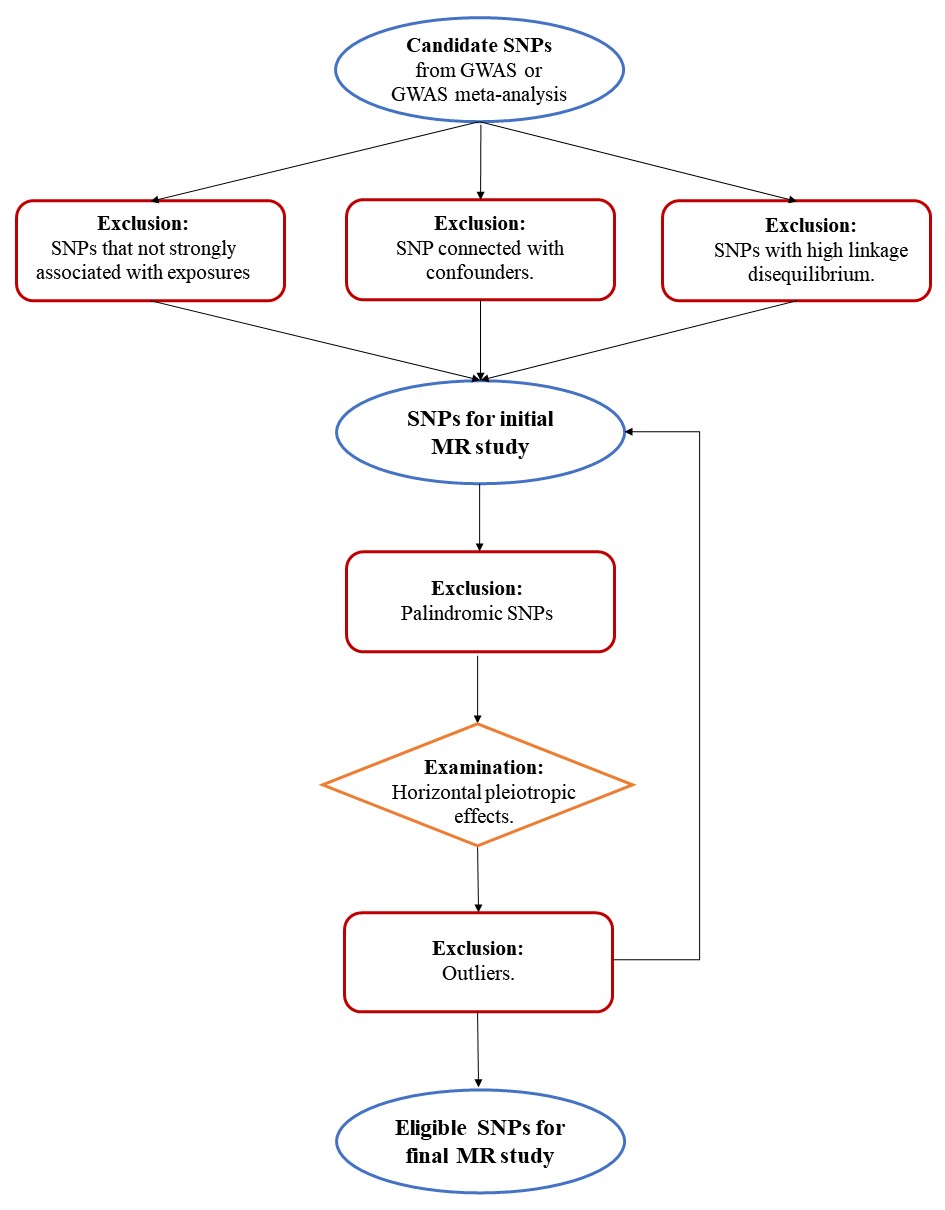

Supplement: Supplementary Figure 1 — The screening and validation process of SNPs: Before initial MR analysis, the candidate SNPs that strongly associated with exposures, not associated with confounders, and with low linkage disequilibrium, were included. In the process of initial MR analysis, for harmonization, the palindromic SNPs were excluded. Subsequently, the horizontal pleiotropic effects were examined to validate whether SNPs can directly affect the outcomes without through the exposure so as to violate the third assumption. Lastly, the outliers that might introduce bias into the results were detected and excluded. After first round of exclusion of ineligible SNPs, the remained SNPs were included for a new round of MR analysis until all ineligible SNPs were excluded, and ultimately the final MR study were performed. [file Image_1.jpeg]
