# Supplementary material for: Genetic associations and potential mediators between psychiatric disorders and irritable bowel syndrome: a Mendelian randomization study with mediation analysis
Source: Front Psychiatry. 2024 Jan 30;15:1279266. doi: 10.3389/fpsyt.2024.1279266 (PMC10861787; doi:10.3389/fpsyt.2024.1279266)
Supplement: Supplementary file 3 [file DataSheet_2.docx]

**Table S10. Associations between genetically predicted potential mediators and IBS.**

| **Potential mediators** | **MR method** | **NSNP** | **OR** | **95%LCI** | **95%UCI** | **P-value** | **Q_P-value** | **MR-Pleiotropy** |
| --- | --- | --- | --- | --- | --- | --- | --- | --- |
| ***Phylum_Actinobacteria*** |  |  |  |  |  |  |  | **0.018** |
|  | MR Egger | 81 | 1.348 | 1.009 | 1.801 | **0.046** | 1 |  |
|  | Weighted median | 81 | 0.929 | 0.898 | 0.961 | **1.933E-05** |  |  |
|  | IVW | 81 | 0.944 | 0.920 | 0.969 | **1.826E-05** | 1 |  |
| ***Family_Streptococcaceae*** |  |  |  |  |  |  |  | 0.905 |
|  | MR Egger | 17 | 1.202 | 0.044 | 33.175 | 0.915 | 1 |  |
|  | Weighted median | 17 | 0.980 | 0.919 | 1.045 | 0.535 |  |  |
|  | IVW | 17 | 0.979 | 0.929 | 1.033 | 0.442 | 1 |  |
| ***Genus_Allisonella*** |  |  |  |  |  |  |  | 0.9985 |
|  | MR Egger | 3 | 0.948 | 0.000 | 78373.111 | 0.994 | 0.932 |  |
|  | Weighted median | 3 | 0.960 | 0.900 | 1.023 | 0.208 |  |  |
|  | IVW | 3 | 0.961 | 0.912 | 1.013 | 0.135 | 0.996 |  |
| ***Genus_Bifidobacterium*** |  |  |  |  |  |  |  | **0.005** |
|  | MR Egger | 249 | 1.179 | 1.065 | 1.306 | **0.002** | 1 |  |
|  | Weighted median | 249 | 0.982 | 0.964 | 1.000 | **0.045** |  |  |
|  | IVW | 249 | 0.997 | 0.984 | 1.011 | 0.705 | 1 |  |
| ***Genus_Enterorhabdus*** |  |  |  |  |  |  |  | 0.982 |
|  | MR Egger | 6 | 1.001 | 0.030 | 33.871 | 1.000 | 0.999 |  |
|  | Weighted median | 6 | 1.047 | 0.981 | 1.118 | 0.169 |  |  |
|  | IVW | 6 | 1.045 | 0.986 | 1.108 | 0.137 | 1 |  |
| ***Genus_Erysipelatoclostridium*** |  |  |  |  |  |  |  | 0.648 |
|  | MR Egger | 3 | 0.025 | 0.000 | 2065.101 | 0.639 | 0.63 |  |
|  | Weighted median | 3 | 0.919 | 0.814 | 1.039 | 0.178 |  |  |
|  | IVW | 3 | 0.895 | 0.810 | 0.988 | **0.028** | 0.73 |  |
| ***Genus_Ruminococcus1*** |  |  |  |  |  |  |  | 0.944 |
|  | MR Egger | 5 | 1.237 | 0.009 | 167.068 | 0.938 | 1 |  |
|  | Weighted median | 5 | 1.020 | 0.909 | 1.144 | 0.736 |  |  |
|  | IVW | 5 | 1.022 | 0.927 | 1.126 | 0.660 | 1 |  |
| ***Genus_Ruminococcustorques*** |  |  |  |  |  |  |  | 0.672 |
|  | MR Egger | 3 | 0.112 | 0.000 | 147.417 | 0.657 | 0.73 |  |
|  | Weighted median | 3 | 0.866 | 0.741 | 1.012 | 0.070 |  |  |
|  | IVW | 3 | 0.888 | 0.780 | 1.012 | 0.074 | 0.8 |  |
| ***Genus_Streptococcus*** |  |  |  |  |  |  |  | 0.05 |
|  | MR Egger | 63 | 1.443 | 0.967 | 2.153 | 0.077 | 1 |  |
|  | Weighted median | 63 | 0.972 | 0.940 | 1.006 | 0.102 |  |  |
|  | IVW | 63 | 0.958 | 0.933 | 0.984 | **0.002** | 1 |  |
| ***Genus_Tyzzerella3*** |  |  |  |  |  |  |  | 0.898 |
|  | MR Egger | 3 | 0.723 | 0.012 | 42.266 | 0.901 | 0.995 |  |
|  | Weighted median | 3 | 1.014 | 0.933 | 1.104 | 0.738 |  |  |
|  | IVW | 3 | 1.012 | 0.942 | 1.087 | 0.748 | 0.987 |  |
| **LPS** |  |  |  |  |  |  |  | 0.45 |
|  | MR Egger | 5 | 0.957 | 0.870 | 1.053 | 0.438 | 0.302 |  |
|  | Weighted median | 5 | 1.007 | 0.954 | 1.062 | 0.811 |  |  |
|  | IVW | 5 | 0.994 | 0.954 | 1.036 | 0.786 | 0.335 |  |
| **Tryptophan** |  |  |  |  |  |  |  | NA |
|  | IVW | 2 | 0.952 | 0.857 | 1.057 | 0.358 | 0.513 |  |
| **Histamine** |  |  |  |  |  |  |  | 0.766 |
|  | MR Egger | 9 | 0.870 | 0.656 | 1.154 | 0.366 | 0.391 |  |
|  | Weighted median | 9 | 0.866 | 0.741 | 1.012 | 0.071 |  |  |
|  | IVW | 9 | 0.905 | 0.803 | 1.020 | 0.104 | 0.486 |  |
| **Acetate** |  |  |  |  |  |  |  | 0.74 |
|  | MR Egger | 6 | 0.561 | 0.132 | 2.383 | 0.477 | **0.01** |  |
|  | Weighted median | 6 | 0.737 | 0.543 | 1.000 | **0.050** |  |  |
|  | IVW | 6 | 0.719 | 0.518 | 0.998 | **0.048** | **0.01** |  |
| **Lactate** |  |  |  |  |  |  |  | 0.404 |
|  | MR Egger | 7 | 0.856 | 0.423 | 1.733 | 0.684 | 0.09 |  |
|  | Weighted median | 7 | 1.126 | 0.926 | 1.370 | 0.233 |  |  |
|  | IVW | 7 | 1.173 | 0.965 | 1.425 | 0.109 | 0.09 |  |
| **Pyruvate** |  |  |  |  |  |  |  | 0.905 |
|  | MR Egger | 19 | 1.053 | 0.872 | 1.272 | 0.596 | **0.037** |  |
|  | Weighted median | 19 | 1.006 | 0.909 | 1.112 | 0.914 |  |  |
|  | IVW | 19 | 1.043 | 0.950 | 1.145 | 0.378 | 0.052 |  |
| **β-Hydroxybutyrate** |  |  |  |  |  |  |  | 0.772 |
|  | MR Egger | 11 | 1.082 | 0.628 | 1.865 | 0.782 | **0.04** |  |
|  | Weighted median | 11 | 1.294 | 1.086 | 1.543 | **0.004** |  |  |
|  | IVW | 11 | 1.171 | 0.997 | 1.375 | 0.054 | 0.06 |  |
| **CRP** |  |  |  |  |  |  |  | **0.02** |
|  | MR Egger | 201 | 0.988 | 0.932 | 1.047 | 0.672 | **0.014** |  |
|  | Weighted median | 201 | 1.018 | 0.959 | 1.080 | 0.559 |  |  |
|  | IVW | 201 | 1.037 | 0.995 | 1.081 | 0.085 | **0.007** |  |
| **IL-6** |  |  |  |  |  |  |  | **4.76E-06** |
|  | MR Egger | 76 | 0.883 | 0.795 | 0.981 | **0.023** | 0.99 |  |
|  | Weighted median | 76 | 1.069 | 1.017 | 1.123 | **0.009** |  |  |
|  | IVW | 76 | 1.137 | 1.102 | 1.173 | **6.923E-16** | 0.542 |  |
| **BDNF** |  |  |  |  |  |  |  | 0.6454 |
|  | MR Egger | 6 | 0.964 | 0.789 | 1.179 | 0.741 | 0.259 |  |
|  | Weighted median | 6 | 1.017 | 0.937 | 1.102 | 0.690 |  |  |
|  | IVW | 6 | 1.011 | 0.946 | 1.081 | 0.745 | 0.346 |  |
| **Catalase** |  |  |  |  |  |  |  | NA |
|  | IVW | 2 | 1.003 | 0.968 | 1.039 | 0.873 | NA |  |
| **Sleep duration** |  |  |  |  |  |  |  | 0.587 |
|  | MR Egger | 10 | 0.860 | 0.183 | 4.052 | 0.854 | **0.00014** |  |
|  | Weighted median | 10 | 0.798 | 0.570 | 1.117 | 0.188 |  |  |
|  | IVW | 10 | 0.916 | 0.546 | 1.537 | 0.740 | **0.00018** |  |
| **Insomnia** |  |  |  |  |  |  |  | **0.021** |
|  | MR Egger | 27 | 34.561 | 3.517 | 339.632 | **0.006** | 0.189 |  |
|  | Weighted median | 27 | 2.053 | 1.292 | 3.264 | **0.002** |  |  |
|  | IVW | 27 | 2.051 | 1.414 | 2.977 | **1.548E-04** | 0.054 |  |
| **BMI** |  |  |  |  |  |  |  | 0.606 |
|  | MR Egger | 258 | 1.018 | 0.874 | 1.185 | 0.820 | **3.08E-05** |  |
|  | Weighted median | 258 | 1.051 | 0.956 | 1.156 | 0.306 |  |  |
|  | IVW | 258 | 1.056 | 0.994 | 1.122 | 0.080 | **3.48E-05** |  |

**Note:** In this table, the potential mediators were exposures, and IBS were outcomes.

NSNP: the number of single nucleotide polymorphism; 95%LCI: the lower limit of 95% confidence interval; 95%UCI: the upper limit of 95% confidence interval; Q_P-value: the results of Q test.
