# Supplementary material for: Genetic associations and potential mediators between psychiatric disorders and irritable bowel syndrome: a Mendelian randomization study with mediation analysis
Source: Front Psychiatry. 2024 Jan 30;15:1279266. doi: 10.3389/fpsyt.2024.1279266 (PMC10861787; doi:10.3389/fpsyt.2024.1279266)
Supplement: Supplementary file 4 [file DataSheet_3.docx]

**Table S35. Associations between genetically predicted psychiatric disorders and potential mediators that significantly associated with IBS**

| **Exposures** | **Potential mediators** | **MR method** | **NSNP** | **OR** | **95%LCI** | **95%UCI** | **P-value** | **Q_P-value** | **MR-Pleiotropy** |
| --- | --- | --- | --- | --- | --- | --- | --- | --- | --- |
| **Broad depression** | ***Genus_Bifidobacterium*** |  |  |  |  |  |  |  | 0.743 |
|  |  | MR Egger | 9 | 3.256 | 0.017 | 615.318 | 0.672 | 0.870 |  |
|  |  | Weighted median | 9 | 1.234 | 0.309 | 4.920 | 0.766 |  |  |
|  |  | IVW | 9 | 1.332 | 0.459 | 3.869 | 0.598 | 0.916 |  |
|  | ***Genus_Erysipelatoclostridium*** | |  |  |  |  |  |  | 0.964 |
|  |  | MR Egger | 9 | 0.649 | 0.001 | 360.287 | 0.897 | 0.704 |  |
|  |  | Weighted median | 9 | 1.180 | 0.201 | 6.939 | 0.854 |  |  |
|  |  | IVW | 9 | 0.752 | 0.204 | 2.765 | 0.668 | 0.795 |  |
|  | ***Genus_Streptococcus*** |  |  |  |  |  |  |  | 0.152 |
|  |  | MR Egger | 9 | 38.090 | 0.278 | 5216.621 | 0.190 | 0.494 |  |
|  |  | Weighted median | 9 | 0.324 | 0.083 | 1.270 | 0.106 |  |  |
|  |  | IVW | 9 | 0.732 | 0.251 | 2.132 | 0.567 | 0.344 |  |
|  | ***Phylum_Actinobacteria*** |  |  |  |  |  |  |  | 0.642 |
|  |  | MR Egger | 9 | 0.723 | 0.004 | 126.210 | 0.905 | 0.297 |  |
|  |  | Weighted median | 9 | 3.384 | 0.925 | 12.386 | 0.066 |  |  |
|  |  | IVW | 9 | 2.526 | 0.924 | 6.905 | 0.071 | 0.368 |  |
|  | **Acetate** |  |  |  |  |  |  |  | 0.402 |
|  |  | MR Egger | 8 | 0.231 | 0.028 | 1.927 | 0.224 | 0.176 |  |
|  |  | Weighted median | 8 | 0.789 | 0.471 | 1.322 | 0.369 |  |  |
|  |  | IVW | 8 | 0.598 | 0.374 | 0.955 | **0.031** | 0.179 |  |
|  | **β-Hydroxybutyrate** |  |  |  |  |  |  |  | 0.353 |
|  |  | MR Egger | 9 | 2.194 | 0.405 | 11.894 | 0.392 | 0.465 |  |
|  |  | Weighted median | 9 | 1.226 | 0.738 | 2.035 | 0.431 |  |  |
|  |  | IVW | 9 | 0.950 | 0.659 | 1.369 | 0.784 | 0.469 |  |
|  | **Insomnia** |  |  |  |  |  |  |  | 0.357 |
|  |  | MR Egger | 9 | 0.888 | 0.374 | 2.104 | 0.794 | **0.025** |  |
|  |  | Weighted median | 9 | 1.374 | 1.136 | 1.660 | **0.001** |  |  |
|  |  | IVW | 9 | 1.356 | 1.126 | 1.633 | **0.001** | **0.02** |  |
|  |  |  |  |  |  |  |  |  |  |
| **MDD** | ***Genus_Bifidobacterium*** |  |  |  |  |  |  |  | 0.482 |
|  |  | MR Egger | 26 | 0.381 | 0.023 | 6.355 | 0.508 | 0.953 |  |
|  |  | Weighted median | 26 | 1.079 | 0.683 | 1.705 | 0.745 |  |  |
|  |  | IVW | 26 | 1.054 | 0.745 | 1.492 | 0.766 | 0.957 |  |
|  | ***Genus_Erysipelatoclostridium*** | |  |  |  |  |  |  | 0.764 |
|  |  | MR Egger | 26 | 1.620 | 0.045 | 58.646 | 0.795 | 0.325 |  |
|  |  | Weighted median | 26 | 1.167 | 0.632 | 2.157 | 0.621 |  |  |
|  |  | IVW | 26 | 0.933 | 0.601 | 1.448 | 0.758 | 0.372 |  |
|  | ***Genus_Streptococcus*** |  |  |  |  |  |  |  | 0.282 |
|  |  | MR Egger | 26 | 4.429 | 0.312 | 62.928 | 0.283 | 0.936 |  |
|  |  | Weighted median | 26 | 1.069 | 0.677 | 1.688 | 0.773 |  |  |
|  |  | IVW | 26 | 1.010 | 0.726 | 1.404 | 0.955 | 0.925 |  |
|  | ***Phylum_Actinobacteria*** |  |  |  |  |  |  |  | 0.576 |
|  |  | MR Egger | 26 | 0.447 | 0.035 | 5.671 | 0.540 | 0.75 |  |
|  |  | Weighted median | 26 | 0.955 | 0.609 | 1.497 | 0.841 |  |  |
|  |  | IVW | 26 | 0.925 | 0.676 | 1.268 | 0.630 | 0.78 |  |
|  | **Acetate** |  |  |  |  |  |  |  | 0.994 |
|  |  | MR Egger | 29 | 1.067 | 0.404 | 2.817 | 0.896 | 0.286 |  |
|  |  | Weighted median | 29 | 1.050 | 0.882 | 1.250 | 0.583 |  |  |
|  |  | IVW | 29 | 1.071 | 0.948 | 1.210 | 0.271 | 0.333 |  |
|  | **β-Hydroxybutyrate** |  |  |  |  |  |  |  | 0.421 |
|  |  | MR Egger | 29 | 1.710 | 0.433 | 6.756 | 0.451 | **0.0001** |  |
|  |  | Weighted median | 29 | 0.899 | 0.757 | 1.069 | 0.230 |  |  |
|  |  | IVW | 29 | 0.969 | 0.813 | 1.154 | 0.721 | **0.0001** |  |
|  | **Insomnia** |  |  |  |  |  |  |  | 0.465 |
|  |  | MR Egger | 27 | 0.984 | 0.538 | 1.798 | 0.958 | **1.94E-10** |  |
|  |  | Weighted median | 27 | 1.092 | 1.009 | 1.181 | **0.028** |  |  |
|  |  | IVW | 27 | 1.234 | 1.141 | 1.334 | **1.40E-7** | **1.72E-10** |  |
|  |  |  |  |  |  |  |  |  |  |
| **Anxiety disorder** | ***Genus_Bifidobacterium*** |  |  |  |  |  |  |  | 0.802 |
|  |  | MR Egger | 5 | 0.862 | 0.283 | 2.624 | 0.810 | 0.707 |  |
|  |  | Weighted median | 5 | 1.023 | 0.918 | 1.140 | 0.680 |  |  |
|  |  | IVW | 5 | 1.006 | 0.920 | 1.099 | 0.900 | 0.832 |  |
|  | ***Genus_Erysipelatoclostridium*** | |  |  |  |  |  |  | 0.428 |
|  |  | MR Egger | 5 | 0.512 | 0.132 | 1.986 | 0.405 | 0.525 |  |
|  |  | Weighted median | 5 | 0.954 | 0.837 | 1.088 | 0.484 |  |  |
|  |  | IVW | 5 | 0.962 | 0.863 | 1.072 | 0.479 | 0.546 |  |
|  | ***Genus_Streptococcus*** |  |  |  |  |  |  |  | 0.498 |
|  |  | MR Egger | 5 | 1.454 | 0.507 | 4.166 | 0.536 | 0.889 |  |
|  |  | Weighted median | 5 | 0.969 | 0.875 | 1.074 | 0.549 |  |  |
|  |  | IVW | 5 | 0.964 | 0.886 | 1.048 | 0.387 | 0.874 |  |
|  | ***Phylum_Actinobacteria*** |  |  |  |  |  |  |  | 0.342 |
|  |  | MR Egger | 5 | 0.503 | 0.134 | 1.895 | 0.385 | 0.158 |  |
|  |  | Weighted median | 5 | 1.091 | 0.973 | 1.223 | 0.136 |  |  |
|  |  | IVW | 5 | 1.076 | 0.964 | 1.200 | 0.190 | 0.116 |  |
|  | **Acetate** |  |  |  |  |  |  |  | 0.919 |
|  |  | MR Egger | 5 | 1.031 | 0.593 | 1.793 | 0.919 | 0.108 |  |
|  |  | Weighted median | 5 | 0.984 | 0.944 | 1.027 | 0.466 |  |  |
|  |  | IVW | 5 | 1.000 | 0.962 | 1.039 | 0.997 | 0.192 |  |
|  | **β-Hydroxybutyrate** |  |  |  |  |  |  |  | 0.319 |
|  |  | MR Egger | 5 | 1.486 | 0.766 | 2.879 | 0.326 | **0.03** |  |
|  |  | Weighted median | 5 | 1.012 | 0.968 | 1.059 | 0.595 |  |  |
|  |  | IVW | 5 | 0.995 | 0.941 | 1.052 | 0.854 | **0.01** |  |
|  | **Insomnia** |  |  |  |  |  |  |  | 0.971 |
|  |  | MR Egger | 5 | 1.003 | 0.805 | 1.249 | 0.981 | **0.033** |  |
|  |  | Weighted median | 5 | 1.008 | 0.993 | 1.023 | 0.273 |  |  |
|  |  | IVW | 5 | 1.007 | 0.992 | 1.023 | 0.351 | 0.069 |  |
|  |  |  |  |  |  |  |  |  |  |
| **PTSD** | ***Genus_Bifidobacterium*** |  |  |  |  |  |  |  | 0.474 |
|  |  | MR Egger | 25 | 1.823 | 0.261 | 12.710 | 0.550 | 0.609 |  |
|  |  | Weighted median | 25 | 0.826 | 0.488 | 1.400 | 0.478 |  |  |
|  |  | IVW | 25 | 0.899 | 0.619 | 1.304 | 0.574 | 0.635 |  |
|  | ***Genus_Erysipelatoclostridium*** | |  |  |  |  |  |  | 0.7 |
|  |  | MR Egger | 25 | 0.709 | 0.058 | 8.612 | 0.790 | 0.297 |  |
|  |  | Weighted median | 25 | 1.172 | 0.612 | 2.246 | 0.632 |  |  |
|  |  | IVW | 25 | 1.154 | 0.718 | 1.856 | 0.554 | 0.341 |  |
|  | ***Genus_Streptococcus*** |  |  |  |  |  |  |  | 0.645 |
|  |  | MR Egger | 25 | 0.656 | 0.085 | 5.035 | 0.689 | 0.191 |  |
|  |  | Weighted median | 25 | 1.133 | 0.687 | 1.870 | 0.625 |  |  |
|  |  | IVW | 25 | 1.056 | 0.717 | 1.555 | 0.783 | 0.222 |  |
|  | ***Phylum_Actinobacteria*** |  |  |  |  |  |  |  | 0.455 |
|  |  | MR Egger | 25 | 1.782 | 0.204 | 15.596 | 0.606 | **0.048** |  |
|  |  | Weighted median | 25 | 0.777 | 0.462 | 1.304 | 0.339 |  |  |
|  |  | IVW | 25 | 0.781 | 0.517 | 1.182 | 0.242 | 0.052 |  |
|  | **Acetate** |  |  |  |  |  |  |  | 0.068 |
|  |  | MR Egger | 29 | 0.552 | 0.341 | 0.893 | **0.023** | 0.83 |  |
|  |  | Weighted median | 29 | 0.833 | 0.701 | 0.990 | **0.038** |  |  |
|  |  | IVW | 29 | 0.867 | 0.767 | 0.979 | **0.021** | 0.709 |  |
|  | **β-Hydroxybutyrate** |  |  |  |  |  |  |  | 0.676 |
|  |  | MR Egger | 29 | 1.220 | 0.738 | 2.015 | 0.445 | 0.309 |  |
|  |  | Weighted median | 29 | 1.138 | 0.949 | 1.363 | 0.162 |  |  |
|  |  | IVW | 29 | 1.098 | 0.969 | 1.245 | 0.142 | 0.348 |  |
|  | **Insomnia** |  |  |  |  |  |  |  | 0.163 |
|  |  | MR Egger | 27 | 1.025 | 0.751 | 1.399 | 0.877 | **4.65E-08** |  |
|  |  | Weighted median | 27 | 1.251 | 1.162 | 1.346 | **2.50E-9** |  |  |
|  |  | IVW | 27 | 1.279 | 1.184 | 1.382 | **4.09E-10** | **7.22E-09** |  |
|  |  |  |  |  |  |  |  |  |  |
| **Schizophrenia** | ***Genus_Bifidobacterium*** |  |  |  |  |  |  |  | 0.991 |
|  |  | MR Egger | 150 | 0.961 | 0.667 | 1.384 | 0.829 | **0.015** |  |
|  |  | Weighted median | 150 | 0.984 | 0.868 | 1.116 | 0.799 |  |  |
|  |  | IVW | 150 | 0.958 | 0.873 | 1.052 | 0.371 | **0.017** |  |
|  | ***Genus_Erysipelatoclostridium*** | |  |  |  |  |  |  | 0.234 |
|  |  | MR Egger | 150 | 0.810 | 0.534 | 1.229 | 0.324 | 0.128 |  |
|  |  | Weighted median | 150 | 0.930 | 0.795 | 1.088 | 0.366 |  |  |
|  |  | IVW | 150 | 1.036 | 0.930 | 1.154 | 0.525 | 0.122 |  |
|  | ***Genus_Streptococcus*** |  |  |  |  |  |  |  | 0.483 |
|  |  | MR Egger | 150 | 1.161 | 0.855 | 1.575 | 0.341 | 0.608 |  |
|  |  | Weighted median | 150 | 0.975 | 0.867 | 1.097 | 0.679 |  |  |
|  |  | IVW | 150 | 1.044 | 0.965 | 1.129 | 0.283 | 0.619 |  |
|  | ***Phylum_Actinobacteria*** |  |  |  |  |  |  |  | 0.785 |
|  |  | MR Egger | 150 | 0.933 | 0.668 | 1.303 | 0.683 | **0.007** |  |
|  |  | Weighted median | 150 | 1.016 | 0.902 | 1.144 | 0.794 |  |  |
|  |  | IVW | 150 | 0.975 | 0.896 | 1.063 | 0.569 | **0.008** |  |
|  | **Acetate** |  |  |  |  |  |  |  | 0.212 |
|  |  | MR Egger | 152 | 0.960 | 0.854 | 1.078 | 0.490 | **0.028** |  |
|  |  | Weighted median | 152 | 1.027 | 0.982 | 1.073 | 0.242 |  |  |
|  |  | IVW | 152 | 1.031 | 0.998 | 1.065 | 0.064 | **0.025** |  |
|  | **β-Hydroxybutyrate** |  |  |  |  |  |  |  | **0.015** |
|  |  | MR Egger | 150 | 1.164 | 1.045 | 1.297 | **0.007** | 0.198 |  |
|  |  | Weighted median | 150 | 0.996 | 0.953 | 1.041 | 0.858 |  |  |
|  |  | IVW | 150 | 1.021 | 0.991 | 1.053 | 0.176 | 0.125 |  |
|  | **Insomnia** |  |  |  |  |  |  |  | 0.416 |
|  |  | MR Egger | 152 | 0.974 | 0.921 | 1.031 | 0.371 | **5.61E-24** |  |
|  |  | Weighted median | 152 | 0.991 | 0.973 | 1.009 | 0.338 |  |  |
|  |  | IVW | 152 | 0.997 | 0.981 | 1.012 | 0.682 | **5.28E-24** |  |

**Note:** In this table, the psychiatric disorders that showed significant causal association with IBS were exposures, and mediators that showed significant causal associations with IBS were outcomes.

NSNP: the number of single nucleotide polymorphism; 95%LCI: the lower limit of 95% confidence interval; 95%UCI: the upper limit of 95% confidence interval; Q_P-value: the results of Q test.
