# Supplementary material for: Genetic associations and potential mediators between psychiatric disorders and irritable bowel syndrome: a Mendelian randomization study with mediation analysis
Source: Front Psychiatry. 2024 Jan 30;15:1279266. doi: 10.3389/fpsyt.2024.1279266 (PMC10861787; doi:10.3389/fpsyt.2024.1279266)
Supplement: Supplementary file 5 [file DataSheet_4.docx]

Table S36: Results of MRlap analysis.

| **Exposure** | **Outcome** | **MRcorrection.corrected_effect** | **MRcorrection.corrected_effect_SE** | **MRcorrection.corrected_effect_*p*** |
| --- | --- | --- | --- | --- |
| Broad depression | IBS | 0.293 | 0.0568 | **2.57E-07** |
| Acetate |  | -0.318 | 0.155 | **0.04** |
| Lactate |  | 0.0356 | 0.0277 | 0.2 |
| Pyruvate |  | 0.00205 | 0.015 | 0.891 |
| β-Hydroxybutyrate |  | 0.233 | 0.1002 | **0.02** |
| Sleep duration |  | -0.0222 | 0.0344 | 0.519 |
| Insomnia |  | 0.177 | 0.0384 | **3.82E-06** |
| BMI |  | 0.00698 | 0.00751 | 0.353 |

**Note:** MRcorrection.corrected_effect, corrected causal effect estimate; MRcorrection.corrected_effect_se, corrected causal effect standard error; MRcorrection.corrected_effect_*p*, corrected causal effect *p*-value. (BMI: body mass index; IBS: irritable bowel syndrome)
