# Supplementary material for: Genetic associations and potential mediators between psychiatric disorders and irritable bowel syndrome: a Mendelian randomization study with mediation analysis
Source: Front Psychiatry. 2024 Jan 30;15:1279266. doi: 10.3389/fpsyt.2024.1279266 (PMC10861787; doi:10.3389/fpsyt.2024.1279266)
Supplement: Supplementary file 6 [file DataSheet_5.docx]

**Table S37: The bias and type 1 error rate in mendelian randomization studies with sample overlap.**

| **Exposure** | **Outcome** | **Bias** | **Type 1 error rate** |
| --- | --- | --- | --- |
| MDD | IBS | 0.006 | 0.05 |
| Anxiety disorder |  | 0.007 | 0.05 |
| PTSD |  | 0.013 | 0.06 |
| AN |  | 0.000 | 0.05 |
| Histamine |  | 0.011 | 0.05 |
| CRP |  | 0.005 | 0.06 |

**Note:** MDD: major depressive disorder; PTSD: posttraumatic stress disorder; AN: anorexia nervosa; CRP: C-reactive protein; IBS: irritable bowel syndrome.
